# Effect of digital game intervention on cognitive functions in older adults: a multiple baseline single case experimental design study

**DOI:** 10.1186/s12877-024-05011-3

**Published:** 2024-05-08

**Authors:** Kyosuke Yorozuya, Yuta Kubo, Keisuke Fujii, Daiki Nakashima, Taiki Nagayasu, Hiroyuki Hayashi, Kazuya Sakai, Keiji Amano

**Affiliations:** 1 Faculty of Rehabilitation and Care, Seijoh University, 2-172 Fukinodai, Tokai, 476-8588 Aichi Japan; 2 Faculty of Health Science, Suzuka University of Medical Science, Suzuka, Mie Japan; 3 Faculty of Health Science, Naragakuen University, Nara, Nara Japan; 4 Good Time Club Grand Hagi, Hagi, Yamaguchi Japan; 5 Faculty of Business Administration, Seijoh University, Tokai, Aichi Japan; 6 College of Image Arts and Sciences, Ritsumeikan University, Kyoto, Kyoto Japan

**Keywords:** Digital game, Single-case design, Cognitive functions, Nursing home, Bayesian analysis

## Abstract

**Background:**

Residents in nursing homes are prone to cognitive decline affecting memory, visuospatial cognition, and executive functions. Cognitive decline can lead to dementia, necessitating prioritized intervention.

**Methods:**

The current study aimed to investigate whether an intervention using a digital game was effective for preserving and improving the cognitive function of residents in nursing homes. An intervention study was conducted using a single-case AB design with multiple baselines. The participants in the study were five older adults aged 65 and over who do not play digital games regularly. The study ran for 15 weeks, including a baseline (phase A) and an intervention phase (phase B). Phase A had five baselines (5 to 9 weeks) with random participant assignment. In phase B, participants engaged in a digital game (Space Invaders) individually. Cognitive function was assessed as the outcome, measured using the Brain Assessment (performed on a tablet through the Internet) at 16 measurement points. Four of five participants (two female and two male) were included in the analysis, using visual inspection and Bayesian statistics with multi-level modeling.

**Results:**

Visual inspection of the graphs revealed cognitive function score improvements after the intervention for most layers in terms of memory of numbers, memory of words, mental rotation test (visuospatial ability), and total scores in the Brain Assessment. These effects were also significant in the analysis by multi-level modeling.

**Conclusions:**

The results suggest that the use of digital games may be effective for preserving and improving cognitive function among residents of nursing home.

**Trial registration:**

This study was registered in the University Hospital Medical Information Network Clinical Trials Registry (UMIN000048677; public title: Effect of a Digital Game Intervention for Cognitive Functions in Older People; registration date: August 30, 2022).

## Introduction

In recent years, the number of people with dementia has reached 55 million globally, and is predicted to increase by approximately 10 million people each year, reaching 131.5 million by 2050; thus, developing solutions for dementia-related problems an important international challenge [1, 2]. Cognitive decline causes behavioral and psychological symptoms of dementia, such as anxiety and wandering, leading to a decline in the quality of life of patients and caregivers. Therefore, measures against cognitive decline are needed to prevent dementia [2, 3]. Residents of nursing homes may be particularly susceptible to cognitive decline because they tend to live in unchanging environments with fewer social activities and less stimulation compared with community-dwelling older adults [4]. Therefore, effective measures are required to maintain and improve cognitive function and prevent the onset of dementia in residents [5]. Specifically, decline in memory, visuospatial ability, and executive functions should be prioritized for intervention, because they reflect the decline in overall cognitive functioning, which can progress into dementia [6–8].

Previous studies have reported that stimulating specific cognitive functions through the implementation of digital games may help maintain cognitive function and prevent dementia among older adults [9, 10]. Digital games are classified as entertainment, and often focus on fun, unlike serious games, which are task-oriented and focus on problem-solving and learning [11]. The operation of digital game controllers involves coordinated movements of the bilateral upper limbs, requiring activation of the cerebellum, which is also associated with procedural and episodic memory [10]. Digital games have the potential to promote the use of visuospatial abilities because of the nature of visuospatial information presented via screens [10, 12]. In addition, the process of processing in-game information and developing operations in real time is likely to facilitate the use of executive functions such as judgment and working memory. Therefore, digital games, which can be easily set up in a facility and are expected to be fun and continuous, may be useful for the prevention of cognitive decline among older adults. However, previous studies have examined the effects of games on improving cognitive function in older adults using experimental designs such as randomized controlled trials. These studies often targeted community-dwelling older adults and utilized interventions involving serious games [10, 13]. However, it has been reported that nursing home residents exhibit different characteristics to those of residents living in the community [14, 15]. Additionally, serious games are typically categorized differently to digital games [11]. Thus, there appears to be a lack of research investigating the effects of digital games on the prevention of cognitive decline, including memory and visuospatial abilities, specifically among nursing home residents. Therefore, at the planning stage of the study, we deemed it necessary to mitigate uncertainty by assessing the feasibility and effectiveness of the research topic before conducting experiments requiring a larger number of participants, such as randomized controlled trials.

To develop effective interventions in situations in which the feasibility and effectiveness of the intervention are uncertain, single-case experimental designs are typically considered to be more appropriate than randomized controlled trial designs that require a large number of participants [12]. In addition, single-case experimental designs with multiple baselines are likely to be more useful for increasing the reliability of the intervention’s impact [16]. The purpose of the current study was to test whether digital games are effective for maintaining and improving global cognitive function, memory, visuospatial ability, and executive function in residents of nursing homes, using a multiple baseline single-case experimental design.

## Methods

This study was conducted according to the Single-Case Reporting Guideline in Behavioural Interventions 2016 Checklist [17].

### Design

This study used a single-case AB design with multiple baselines, with phase A as the no-intervention phase and phase B as the intervention phase. This study was registered in the University Hospital Medical Information Network Clinical Trials Registry (UMIN000048677; public title: Effect of a Digital Game Intervention for Cognitive Functions in Older People; registration date: August 30, 2022).

### Setting and study population

In this study, five residents in one nursing home facility, were selected as participants between November 1, 2022, and March 31, 2023. Inclusion criteria were as follows: (1) 65 years of age or older　(on the basis of previous studies, the older adults in this study were defined as individuals aged 65 years or older) [18, 19], (2) Mini Mental State Examination-Japanese (MMSE-J) [20, 21] score of 24 points or higher (maximum score: 30 points), (3) Ability to perform the test and tasks in the questionnaire. (4) Ability to operate a game controller without hand impairment, and (5) No regular habit of playing games (less than once a month), such as Nintendo Switch and smartphone-based games on a regular basis. Exclusion criteria were as follows: (1) severe behavioral disorders or medical requirements (e.g., a person whose sitting time is limited because of a disease such as heart failure, and who might be negatively impacted by the game intervention), (2) severe visual or hearing impairments, and (3) refusal to participate in the study.

### Data collection

The minimum number of cases required for a single-case design with multiple baselines is three or more [16]. Several intervention studies with single-case designs that have been validated with three or four cases have previously been reported [12, 22]. Therefore, we sought to include at least four cases in the current study, and the number of participants was set at 5 to take account of a potential dropout rate of 20%. The study collaborators collected information on basic characteristics such as age, gender, marital status (married, widowed, divorced, single), educational attainment (elementary school, junior high school, high school, university), length of stay, locomotion, and Barthel index from the facility records.

### Digital game intervention

“Space Invaders” was used for the intervention in phase B of this study. Space Invaders is a two-dimensional (2D) platform digital game developed by Taito Corporation in 1978, which became popular worldwide (https://spaceinvaders.jp/whats.html). In Space Invaders, the aim is to shoot and eliminate the army of enemy aliens (Invaders) approaching from the top of the screen, using a beam cannon that can be moved left or right with the controller. When the invaders reach the player’s position at the bottom of the screen, the words “GAME OVER” are displayed, and the game ends. The game is completed when all invaders are eliminated before they reach the player’s position at the bottom of the screen, and the game proceeds to the next stage. The difficulty level increases as the game is cleared and progresses. If the “GAME OVER” message appears, the game can be restarted from the first stage. In the case of digital games that require complex controller-operation, older adults are likely to experience difficulty because of age-related loss of dexterity [23], which may interfere with continued implementation. Space Invaders can be easily operated with the left and right cross keys and a single right button, and the rules are easy to understand; it is classified as an entertainment game, which focuses on enjoyment, in contrast to task-oriented serious games that focus on the problem-solving and learning of the implementer [11]. In addition, because Space Invaders was a particularly popular game in the 1970s, we speculated that prior knowledge of the game among older adults (eliciting a sense of familiarity such as “I have seen it before” or “I know it”) might also contribute to continued engagement and enjoyment of the game. We speculated that this software would be suitable for this purpose because it is familiar to many older adults, has simple rules, and is linked to enjoyment and ongoing engagement. The game console used was the Nintendo switch and its dedicated family computer controller.

**Fig. 1:**
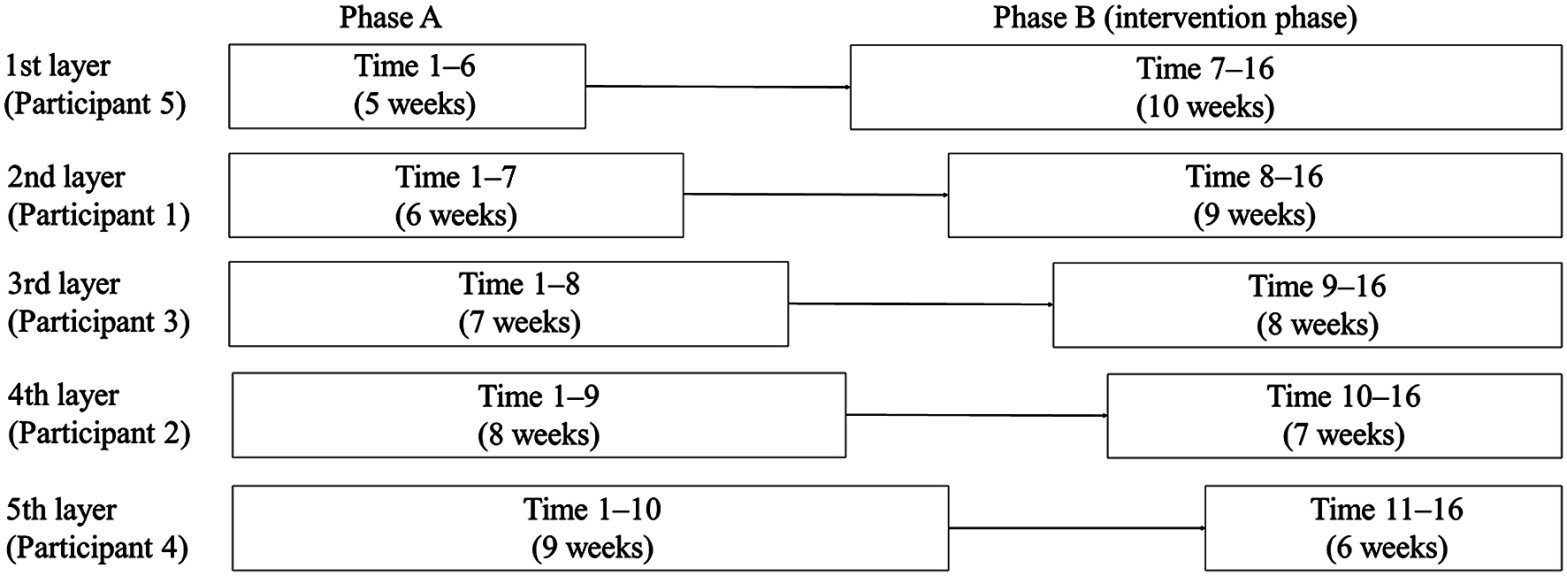
Assigning participants to each layer. Participants 1, 2, 4, and 5 underwent the Brain Assessment once a week, for a total of 16 times, including the first time. The time required to conduct the Brain Assessment was 10 min each time (five tasks each requiring 2 min). Participants 1, 2, 4, and 5 underwent digital intervention for 27, 21, 18, and 30 sessions, respectively, for 30 to 60 min, three times a week during phase B. Participant 3 left the study midway through the study, so only underwent Brain Assessment a total of eight times, including the first time, and did not undergo the digital intervention

The duration and frequency of digital game playing ranged from 30 to 60 min per session, three times per week [10]. For the intervention environment, a private room for game playing was set up in the cooperating nursing home facility, and a staff member at the facility, who was a collaborator in the study, guided the participants to the room in which the game was played. After the induction, the participants played the game alone, during which time they had as little communication with others as possible. Digital games were played during the time in which participants would normally watch television or engage in hobbies or other activities. Instruction regarding the connection and operation of the TV monitor and Nintendo Switch (week 1 of phase B) was provided by staff. During the first week, all participants were able to understand and manipulate the rules of the game, and the staff confirmed that each participant was able to play the game alone to the same degree. The study period was 15 weeks (16 measurement points), with phases A and B divided into five layers (5 and 10 weeks, 6 and 9 weeks, 7 and 8 weeks, 8 and 7 weeks, and 9 and 6 weeks), and participants were randomly assigned to each layer. On the basis of previous studies with cognitive function as the outcome, the minimum duration of intervention in phase B was 6 weeks [24]. The phase A was defined as the period during which participants continued their usual lifestyle (Fig. 1).

### Outcome measure

**Fig. 2:**
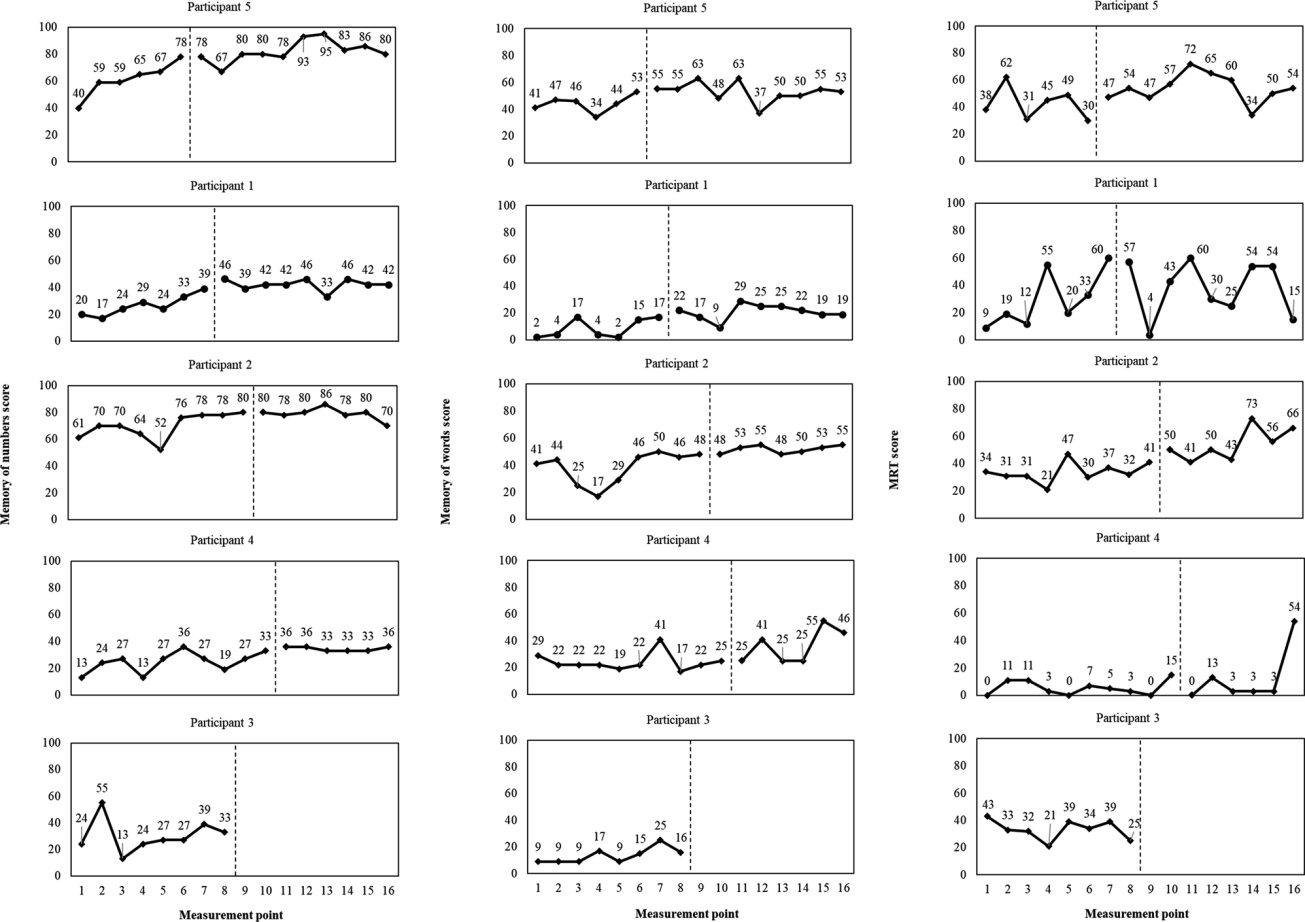
Progress of each Brain Assessment item (memory of numbers, memory of words, and MRT) score. The left side of the vertical line represents phase A, while the right side represents phase B. MRT, mental rotation test

**Fig. 3:**
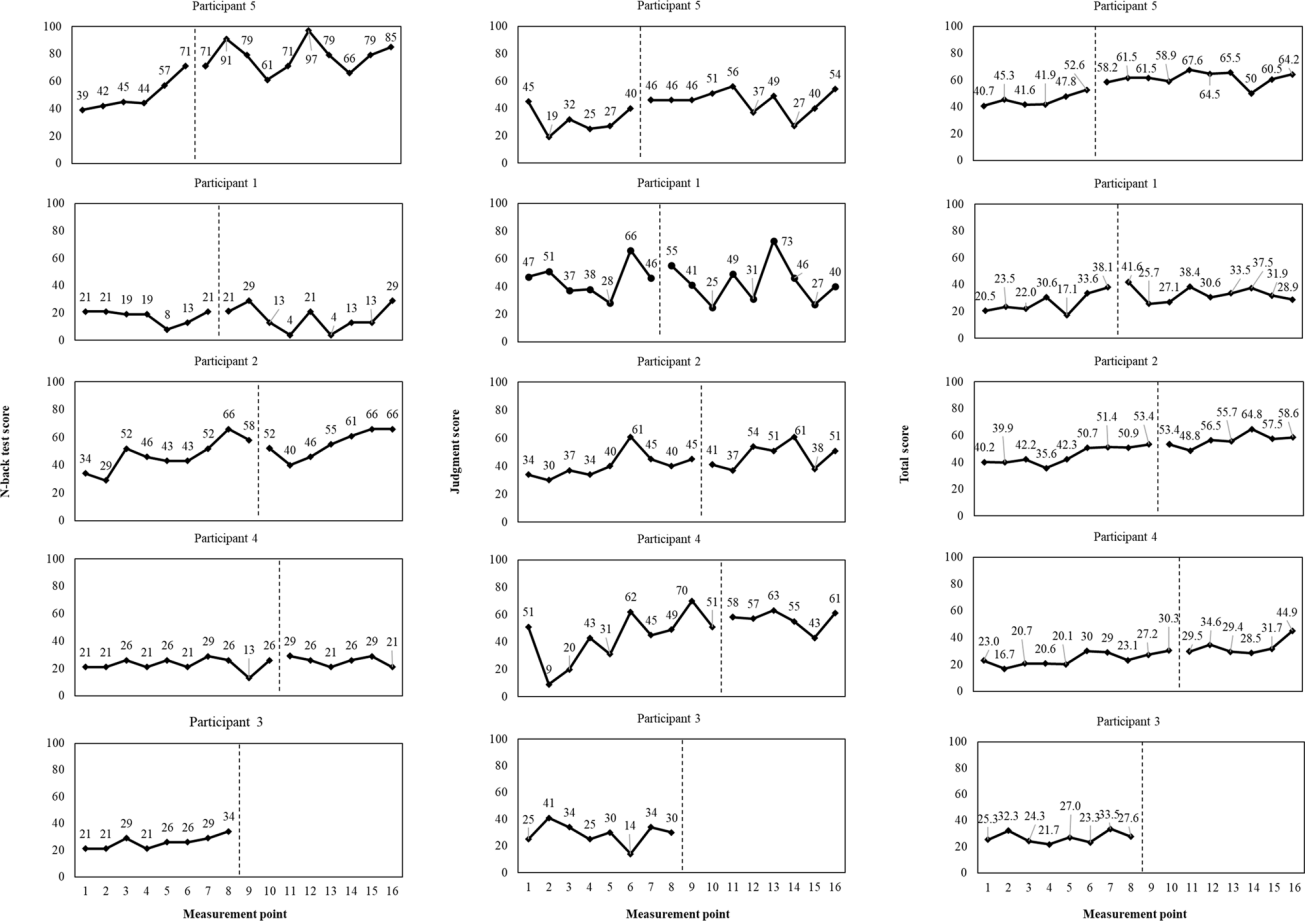
Progress of each Brain Assessment item (N-back test, judgment, and total) score. The left side of the vertical line represents phase A, while the right side represents phase B

The Brain Assessment instrument was used to assess specific and global cognitive functions. Brain Assessment consisted of five subsets, as follows. (1) Memory of numbers: participants were instructed to memorize numbers consisting of three to nine digits, and repeat them using the numeric keypad in ascending and descending order. (2) Memory of words: participants were instructed to memorize five target words. After a short distraction task, 10 words, half of which belong to the target word, were presented in turn. Participants reported whether each word was included in the target word. (3) Mental rotation test (MRT) to assess visuospatial ability: a target block diagram was displayed. One of the four rotated choices was different from the target. Participants chose which option was different. (4) N-back test to assess working memory: a series of numbers appeared, and participants performed the addition of the present and some antecedent numbers. (5) Judgment task: participants were instructed to identify changes in the presented Figs. 2 and 3. Cognitive scores were calculated for each subset and total of each subset, allowing the state of the participant’s cognitive status to be assessed. Cognitive score is calculated as (raw score) – (mean of the raw score)/(standard deviation of the raw score) × 10 + 50. Higher cognitive score indicates higher cognitive function (by the same age and sex [mean and standard deviation are based on data from 5,000 subjects]). The validity and reliability of this measure have been confirmed [25–27]. The time required for each item was 2 min (a total of 10 min for five items), and the target age group was 40–90 years old. Each task is randomly assigned, and the results are not fed back, so the effect of learning through repetition is minimal. Each participant underwent the Brain Assessment individually using a tablet connected to the Internet. Assessments were conducted at the same time each week. During the study period, the survey was conducted approximately once a week from the time of participant selection to the end of follow-up (16 times in total). The total cognitive score in the Brain Assessment indicated global cognitive function, and was determined by the results of each cognitive score (five subsets) in the Brain Assessment. Therefore, the primary outcome of the intervention was assumed to be the cognitive score for each subset in the Brain Assessment, and the secondary outcome was assumed to be the total cognitive score in the Brain Assessment.

### Statistical analysis

The single-case experimental design has a hierarchical structure in which the measurement period is tied to the study participants (the measurement period is nested by the study participants). In this study, we applied a two-level multilevel model with the measurement period as level 1 and the study participants as level 2 [28, 29]. In addition, there were five participants in this study, and the number of data points was 16 points per participant, or 80 points for five participants. It was assumed that the sample size would not be large enough to take into account the possibility of drop-outs. Therefore, the analysis used Bayesian modeling, which can be used with small sample sizes [28, 29].

In the modeling, the objective variable (each cognitive score of the Brain Assessment) was considered as a continuous variable, and phase was a dummy variable with phase A = 0 and phase B = 1. The parameters are denoted by ${mu}_{0}$ for the average baseline value and ${mu}_{1}$ for the average intervention effect. Variation among participants is indicated by ${sigma}_{0}$, ${sigma}_{1}$. The sum of ${mu}_{0}$ and ${mu}_{1}$is the average value of phase B. The results are presented as expected a posteriori (EAP) and 95% Bayesian confidence interval (CI). In Bayesian statistics, if the 95% Bayesian CI of ${mu}_{1}$, the parameter of the intervention effect, does not contain 0, 0 is judged to not be a possible value of the parameter, and the result is interpreted as significant [28].

Bayesian estimation and the Markov chain Monte Carlo (MCMC) method were used for parameter estimation, and MCMC sampling was conducted 18,000 times (chain = 3, burn-in = 1000). In the convergence judgment, MCMC was judged to have converged to a steady state when the potential scale reduction factor (PSRF) < 1.05 [30]. The statistical software R (version 4.0.5; R Foundation for Statistical Computing, Vienna, Austria) with the runjags (version 2.2.0–2) package and rjags (version 4–10) package were used for all statistical analyses.

## Results

### Demographic characteristics

Of the five participants, two were female. The ages of participants 1–5 were 74, 86, 89, 90, and 65 years, respectively. MMSE-J scores for participants 1–5 were 24, 25, 26, 27, and 25, respectively (Table 1). All participants were independently ambulant, and all achieved a perfect score of 100 points on the Barthel Index, which serves as an indicator of activities of daily living (ADL). Participants 1, 2, 4, and 5 carried out the entire intervention, with a final implementation rate of 100% (Participant 1, 27/27; Participant 2, 21/21; Participant 4, 18/18; Participant 5, 30/30). Participant 3 was discharged and dropped out before the intervention.

**Table 1:** Characteristics of the study participants

	P1	P2	P3	P4	P5
Age, years	74	86	89	90	65
Sex	Male	Female	Male	Male	Female
Education	High school	High school	Junior high school	Junior high school	High school
Length of stay, months	21	23	21	20	2
Marital status	Single	Widowed	Widowed	Widowed	Married
Locomotion	Independent gait	Independent gait	Independent gait	Independent gait	Independent gait
BI, score	100	100	100	100	100
MMSE-J, score	24	25	26	27	25
Brain Assessment, score					
Memory of numbers	20.0	61.0	24.0	13.0	40.0
Memory of words	2.0	41.0	9.0	29.0	41.0
MRT	9.0	34.0	43.0	0.0	38.0
N-back test	21.0	34.0	21.0	21.0	39.0
Judgment	47.0	34.0	25.0	51.0	45.0
Total	20.5	40.2	25.3	23.0	40.7

BI, Barthel index; MMSE-J, Mini Mental State Examination-Japanese; MRT, mental rotation test Results of visual inspection of graphs

No adverse events were observed in any of the participants throughout the study period. The results in Figs. 2 and 3 were determined through visual inspection [12]. Because participant 3 moved out at the 8-week mark and the intervention could not be completed, only phase A was assessed. In the layers of memory of numbers, memory of words, and total scores, cognitive scores appeared to gradually improve in all participants from the start of the intervention. In the MRT, cognitive scores appeared to gradually improve in Participants 2 and 5 from the start of the intervention. In the case of Participant 1, there was significant variability, and no apparent difference between phase A and phase B was observed. In Participant 4’s MRT performance, cognitive scores appeared to remain relatively stable throughout the study, but an improvement was observed towards the end. For the N-back test, Participant 5 exhibited an improvement in cognitive scores following the intervention, which appeared to be quite diverse. However, other participants displayed consistent fluctuations. In the judgment task, there was a minimal difference between phase A and phase B for all participants (Figs. 2 and 3).

### Results of estimates for Brain Assessment using multilevel model

An analysis was conducted using a multi-level model with Bayesian statistics for the remaining four participants after excluding Participant 3, who dropped out. The MCMC results indicated that all posterior distributions converged to a steady state (PSRF < 1.05). Therefore, the posterior distribution was considered to be appropriate for use a probability distribution.

In the estimated results for the intervention effect, ${mu}_{1}$, significant effects were observed in memory of numbers (EAP = 13.50, [95% Bayesian CI: 5.22, 21.40]), memory of words (EAP = 11.50, [95% Bayesian CI: 8.48, 14.43]), MRT (EAP = 11.57, [95% Bayesian CI: 2.70, 20.46]), and total score (EAP = 10.61, [95% Bayesian CI: 3.86, 16.98]) (Table 2). The N-back test and judgment task did not reveal significant intervention results.

**Table 2:** Effect of digital game intervention on brain assessment (Bayesian multilevel model analysis)

	Memory of numbers			Memory of words			MRT		
	EAP (SD)		95% Bayesian CI	EAP (SD)		95% Bayesian CI	EAP (SD)		95% Bayesian CI
mu0	36.42	(15.50)	[0.19, 62.70]	25.59	(10.69)	[1.38, 44.76]	25.21	(10.63)	[1.48, 44.29]
mu1	13.50	(4.00)	[5.22, 21.40]	11.50	(1.54)	[8.48, 14.43]	11.57	(4.62)	[2.70, 20.46]
phase B	49.91	(16.00)	[12.37, 77.40]	37.09	(10.82)	[12.95, 56.43]	36.78	(11.58)	[11.10, 57.98]
sigma0	32.34	(21.19)	[13.29, 83.34]	21.04	(12.73)	[9.03, 55.19]	20.92	(12.55)	[8.94, 54.16]
sigma1	7.33	(4.98)	[3.00, 19.84]	2.65	(1.92)	[1.09, 7.12]	8.14	(5.46)	[3.36, 22.08]
	**N-back test**			**Judgment**			**Total**		
	**EAP (SD)**		**95% Bayesian CI**	**EAP (SD)**		**95% Bayesian CI**	**EAP (SD)**		**95% Bayesian CI**
mu0	30.19	(11.57)	[3.77, 50.32]	38.99	(5.09)	[27.72, 47.49]	32.81	(8.83)	[11.57, 47.78]
mu1	8.54	(8.68)	[− 9.37, 25.82]	7.72	(5.50)	[− 3.76, 18.43]	10.61	(3.28)	[3.86, 16.98]
phase B	38.74	(14.42)	[6.30, 64.49]	46.71	(7.52)	[30.20, 61.46]	43.42	(9.40)	[21.38, 59.73]
sigma0	22.17	(13.76)	[9.31, 56.03]	8.35	(5.85)	[3.36, 22.43]	15.85	(10.63)	[6.53, 43.44]
sigma1	17.24	(10.56)	[7.40, 43.34]	9.78	(6.12)	[4.08, 25.27]	5.66	(3.94)	[2.31, 14.84]

MRT, mental rotation test; EAP, expected a posteriori; SD, standard deviation; 95% Bayesian CI, 95% Bayesian confidence interval

## Discussion

The current study examined the effects of an intervention using a digital game (Space Invaders) on the cognitive function of five residents in nursing home. A single-case AB design with a multiple baseline approach was employed for this investigation. The results shown in Figs. 2 and 3 indicate a gradual improvement in cognitive scores for memory of numbers, memory of words, and total scores among all participants following the intervention. Most participants exhibited stable or improved performance after the intervention, suggesting that these changes may be attributed to the implementation of Space Invaders. In the analysis results, cognitive scores for memory of numbers, memory of words, and total scores were higher in phase B compared with those in phase A, indicating a significant intervention effect. This result supports the potential impact of playing Space Invaders observed through visual inspection. However, some participants showed a gradual improvement in cognitive scores even before the intervention, suggesting the involvement of residual variables other than playing the game. In the MRT, participant 1 exhibited substantial variability, indicating a lack of expected game-related effects. In contrast, other participants showed potential for improvement, and as a result, the analysis suggested a significant intervention effect.

The results for memory of numbers, memory of words, and the MRT, which reflect memory and visuospatial ability, appeared to support the hypothesis of this study [26]. We speculated that controller operation would promote cerebellar activity related to memory, and that processing information from the screen during the game would involve visuospatial cognitive abilities. These potential effects might be expected to occur in 2D platform digital games like Space Invaders [9]. Furthermore, the finding that simultaneous effects were observed for memory and visuospatial ability, both of which are considered to be crucial for the prevention of cognitive decline, suggests that the implementation of interventions involving Space Invaders may have potential for the prevention of cognitive decline [6–8]. Moreover, 2D platform digital games are easily recognizable in terms of controls and screen movements, potentially contributing to their consistent adherence (100% implementation rate). These aspects serve as advantages of this intervention and are likely to be factors contributing to its effectiveness. The significant improvement in total cognitive scores, reflecting global cognitive function, suggests that the use of 2D platform digital games like Space Invaders should be considered as a potential strategy in future efforts to prevent cognitive decline. Therefore, exploring the utilization of games like Space Invaders as a means of cognitive intervention for the prevention of cognitive decline should be encouraged.

Interventions related to executive functions, such as the N-back test and judgment task [31, 32] did not reveal significant improvements. This might suggest the need for activities that involve three-dimensional (3D) environments or games that require predicting the surrounding space in a more 3D manner and strategizing [11]. A previous study using the Wii Fit® game console, a 3D platform, reported that after one session, the semantic memory and executive function of nursing home residents improved moderately [33]. A study examining the effects of the Wii Fit® game console on cognitive function in older adults living in a nursing home or attending a day care center reported improvements in global cognitive function [34]. Beyond the findings of the current study, it is crucial to continue researching the features of more effective games, including those with 3D platforms, competitive elements, or role-playing aspects.

The estimation results for each cognitive score in the Brain Assessment exhibited wide confidence intervals, suggesting that the dispersion of the estimated averages in the analysis was large. In this study, we targeted five participants, which was considered to be the minimum number of participants using a single-case design. However, one participant dropped out before the intervention, and the actual analysis was performed on four patients. Although the participants were selected using specific criteria, there was a large amount of variation in the cognitive scores of each participant, which may have affected the accuracy of the estimation. To further improve the accuracy of estimation in future studies, it will be necessary to secure more participants and verify the effectiveness of this method.

Although the current findings suggest several areas for further investigation, Space Invaders, as implemented in this study, demonstrated high practical utility, with a 100% implementation rate and no adverse events. Notably, the current findings indicated that residents of a nursing home facility were able to engage in this intervention independently.

This research paradigm was designed to examine the feasibility and effectiveness of a game-based intervention. Compared with the designs of some previous studies, such as randomized controlled trials, the ability of the current study design to confirm a potential effect on a population is relatively limited. However, to the best of our knowledge, no previous studies have specifically considered the effects of 2D digital games on preventing decline in cognitive functions such as memory and visuospatial abilities in nursing home residents. The current findings suggested the feasibility and effectiveness of an intervention using Space Invaders for preventing cognitive decline in nursing home residents, which may have implications for future research. In the future, higher-quality study designs should be used to confirm the current findings.

## Limitations

However, some limitations of the current study should be considered. We found that some participants showed gradual improvements (covariation) in cognitive scores, even before the intervention. The Brain Assessment is considered to be less susceptible to learning effects because the task is presented with a choice of five versions, and no feedback regarding correct answers is provided to the test taker [26]. However, it is possible that the changes in the Brain Assessment in phase A in this study resulted from habituation caused by repeated administration of the test, and such a learning effect might be unavoidable. The current study design could not conclusively confirm the presence or impact of these residual variables. However, because the analysis also accounted for the effects of variables that are difficult to observe (such as learning effects) by incorporating random effects as level 2 for participant-specific variation, it is possible that the significant results for post-intervention cognitive score variation included intervention effects. In future research, it will be essential to develop more extended study plans that consider factors like seasonal variations and introduce interventions after ensuring the stability of fluctuations observed in the phase A. In addition, larger sample sizes will be needed to test the effects of digital game interventions.

In this study, the duration of the intervention varied among participants, and the possible influence of this variation on the observed effects cannot be excluded. In future studies, it will be important to standardize the intervention period for all participants and to unify the amount of intervention. Moreover, employing more robust research designs, such as randomized controlled trials, will be crucial for verifying the effects identified in the current study.

## Conclusions

The Space Invaders intervention implemented in this study appears to have potential for enhancing memory of numbers, memory of words, MRT performance, and total cognitive scores for residents of nursing home. The high practicality of this intervention, which can be independently used by individuals and has no reported adverse events, further supports its utility in real-world settings. The effectiveness of this intervention should be tested in more detail in future, higher-quality research.

## Data Availability

All data generated or analysed during this study are included in the manuscript.

## Abbreviations

MMSE-J: Mini Mental State Examination-Japanese
2D: two-dimensional
MRT: Mental rotation test
EAP: expected a posteriori
CI: confidence interval
MCMC: Markov chain Monte Carlo
PSRF: potential scale reduction factor
ADL: activities of daily living
SD: standard deviation
3D: three-dimensional
